# Health practitioners' readiness to address domestic violence and abuse: A qualitative meta-synthesis

**DOI:** 10.1371/journal.pone.0234067

**Published:** 2020-06-16

**Authors:** Kelsey Hegarty, Gemma McKibbin, Mohajer Hameed, Jane Koziol-McLain, Gene Feder, Laura Tarzia, Leesa Hooker

**Affiliations:** 1 Department of General Practice, University of Melbourne, Melbourne, Victoria, Australia; 2 The Royal Women’s Hospital, Melbourne, Victoria, Australia; 3 School of Clinical Sciences, Auckland University of Technology, Auckland, New Zealand; 4 Bristol Medical School, University of Bristol, Bristol, United Kingdom; 5 Judith Lumley Centre, La Trobe University, Bundoora, Victoria, Australia; University of Michigan, UNITED STATES

## Abstract

Health practitioners play an important role in identifying and responding to domestic violence and abuse (DVA). Despite a large amount of evidence about barriers and facilitators influencing health practitioners’ care of survivors of DVA, evidence about their readiness to address DVA has not been synthesised. This article reports a meta-synthesis of qualitative studies exploring the research question: What do health practitioners perceive enhances their readiness to address domestic violence and abuse? Multiple data bases were searched in June 2018. Inclusion criteria included: qualitative design; population of health practitioners in clinical settings; and a focus on intimate partner violence. Two reviewers independently screened articles and findings from included papers were synthesised according to the method of thematic synthesis. Forty-seven articles were included in the final sample, spanning 41 individual studies, four systematic reviews and two theses between the years of 1992 and 2018; mostly from high income countries. Five themes were identified as enhancing readiness of health practitioners to address DVA: *Having a commitment; Adopting an advocacy approach; Trusting the relationship; Collaborating with a team; and Being supported by the health system*. We then propose a health practitioners’ readiness framework called the CATCH Model (Commitment, Advocacy, Trust, Collaboration, Health system support). Applying this model to health practitioners’ different readiness for change (using Stage of Change framework) allows us to tailor facilitating strategies in the health setting to enable greater readiness to deal with intimate partner abuse.

## Introduction

Global policies state the urgent need to address domestic violence and abuse (DVA).This ‘wicked chaotic problem’ [1] demands a complex inter-sectoral approach underpinned by a strong universal health system capacity to identify and tailor responses to the circumstances of affected families. The World Health Organisation (WHO) has identified the crucial role of an effective health system in reducing the extensive damage from DVA, especially for children. [2–4] DVA has a high prevalence with a major impact on the health and wellbeing of women, men, children, wider family networks and society as a whole. Globally, one in three women experience physical or sexual violence by partners. [2] DVA damages the mental and physical health of individual women, men, young people and children [2] and is a leading contributor to disease burden for women of child bearing age. [5] Women are more likely than men to experience severe physical, emotional and sexual abuse from a current or past partner, causing fear, injuries, and death. [2] The illness and suffering among survivors of DVA and their children is substantial and results in increased use of medical services and loss of days worked. [2]

Health services have lagged behind other agencies in responding to DVA, [4] despite the fact that the majority of families experiencing DVA frequently attend health services. [2] General practice, antenatal clinics, community child health and emergency departments are key places for intervention for DVA, as health practitioners are the major professional group to whom patients want to disclose. [6] Only a minority of women, men and/or children exposed to DVA are recognised in health care settings. [4] However, we know that patients want to be asked directly about DVA by supportive practitioners, typically making multiple visits before disclosure. [6] Unfortunately, when patients do disclose, there is evidence that health professionals often lack the essential skills and experience to respond appropriately. [3] Much less is known about health practitioners’ capacity to identify and respond to children exposed to DVA or to men who experience or use violence in their intimate relationships. [7]

Despite a wealth of studies exploring the barriers and facilitators to identification and response to DVA, there remain major gaps in knowledge regarding the best ways to support and train health practitioners to enable an evidence-based pathway to safety for family members through the health system. [3, 6, 8–11] Literature has mostly focused on inquiry about DVA and disclosure revealing low rates with one third of women who have experienced DVA ever disclosing, and an inquiry rate by practitioners of between 10–30 percent. [3, 12] Further, evidence has mainly focused on barriers to patients disclosing (shame, being judged or not believed, and confidentiality concerns) or barriers for health practitioners’ identification (insufficient time or skills, feeling overwhelmed by the emotional nature of the work or their own DVA experience) or facilitators to identification (information, screening tools, skills training, support). [4, 10] However to understand in depth what enables health practitioners to undertake this complex work of addressing DVA, we need to look more closely at what makes health practitioners ready to address the complex issue of DVA. The concept of ‘*readiness*’ has been described as a positive force that may motivate people to make positive changes [13, 14] and can include self-efficacy, emotions, motivations and attitudes. Readiness is not only just describing the facilitators to the work or how to overcome the barriers. These facilitators are often seen as ‘*preparedness*’ through increasing knowledge and skills of health practitioners but readiness goes beyond this state, with practitioners physically and emotionally ready for the work. [15] To capture the complexity of practitioners’ voices about their readiness, we focused on qualitative study findings. [6] Thus, to fill the gap in the literature, our aim was to explore health practitioners’ perceptions of what enhances their readiness to address intimate partner violence.

## Method

A standard approach to conducting a qualitative meta-synthesis was adopted. [16] The synthesis involved several stages: (i) formulating a research question; (ii) undertaking a systematic search of the evidence; (iii) screening studies in accordance with inclusion and exclusion criteria; (iv) extracting data from the included studies into data extraction forms; (v) assessing the quality of the included studies; (vii) synthesising the findings from the studies; and (vi) assessing the quality of the findings that emerged from the synthesis.

### Search strategy

Our search strategy was guided by our research question: What do health practitioners perceive enhances their readiness to address intimate partner violence? Seven bibliographic databases were searched: MEDLINE; EMBASE; CINAHL; PsychINFO; SocINDEX; ASSIA and the Cochrane Library. No time limits were applied to the search. The search involved three platforms: Ovid; Ebsco; and ProQuest. The Ovid search was designed using subject headings, keywords and text words for the categories: intimate partner violence; qualitative research; health practitioners. The Ovid search strategy was then translated into language appropriate for the Ebsco and ProQuest platforms. Although some terms differed slightly between platforms, the meaning of each search was preserved across each. For example, the phrase “social sciences/ or theoretical orientation” was translated from Ovid to Ebsco as “MH social science or ‘social science*’ or MH conceptual framework,” and then to ProQuest as “mainsubject” (social sciences). Grey literature was searched for via databases GreyLit and OpenGrey, as well as the first 60 results in a Google Scholar search. The database search was complemented by discussions with experts in the field (see S1 Table for search terms).

### Study selection

**Fig 1:**
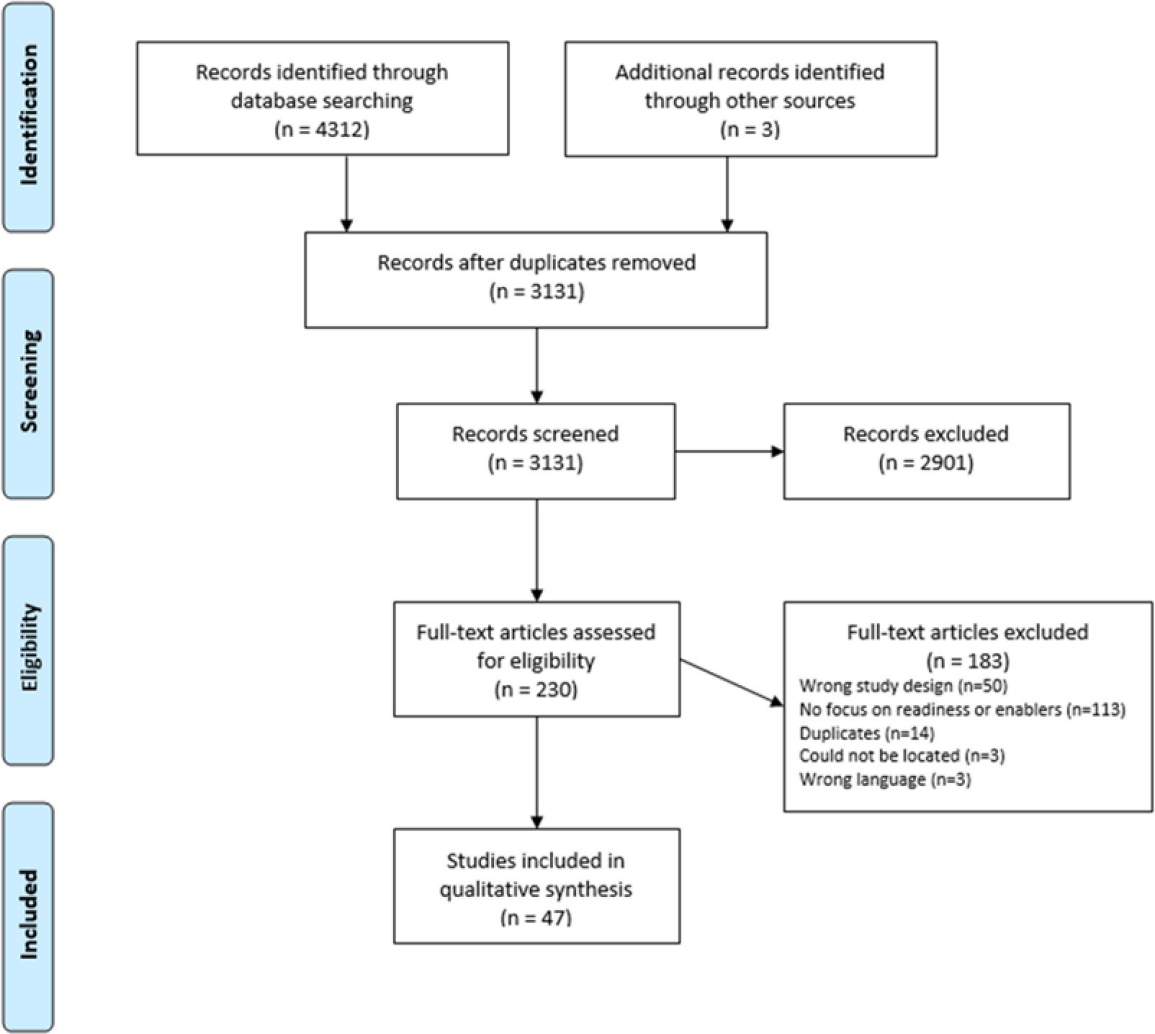
Flowchart of study selection.

The database search generated 4,312 results and three further records were identified from experts in the field (see Fig 1). The records were imported into Covidence [17] a program to assist with study selection for reviews. Two reviewers (GM and MH) undertook title and abstract screening applying the following inclusion criteria: (1) a qualitative data collection and analysis method; (2) a mixed-methods design if separate qualitative data collection and analysis findings presented; (3) a population of doctors, nurses, midwives, allied health professionals or Aboriginal health workers; and (4) a focus on intimate partner violence (survivors, perpetrators, children exposed). Studies were excluded under the following conditions: (1) written in a language other than English; (2) a population of social workers, health managers or students only; (3) a focus on child abuse or adolescent family violence; and (4) a focus on barriers to addressing DVA only.

The same two reviewers carried out full text screening through applying the inclusion and exclusion criteria but also through applying a further criterion. Studies were included that had either an explicit focus on readiness or facilitators that enhance health practitioners’ response to intimate partner violence or referred to readiness or facilitators as part of exploring experiences of health practitioners addressing intimate partner violence. Disagreements between reviewers were resolved through discussion and a third reviewer (KH) checked the final sample against the inclusion and exclusion criteria. Forty-seven articles met the criteria and were included in the review (see Table 1).

**Table 1:** Study and participant characteristics.

Source	Country	Objective	Method	Theories	Setting	Sample (Gen, #, prof, age)	Years of clinical experience	Training received
Allen et al., 2011	USA	To compare providers and health care settings at two points in time to explore the degree to which the Health Care Council achieved proximal outcomes in the health care response to DVA	Mixed methods Focus groups	Grounded Theory	Primary Emergency Obstetrics/gynaecology Intensive care	**19** Practitioners 100% women	Not stated	100%
Al-Natour et al., 2016	Jordan	To describe Jordanian nurses’ roles and practices in screening for DVA.	Qualitative Semi-structured interviews	Phenomenology	Emergency	**12** Nurses 50% women 50% men	Mean 7 years	Not stated
Baig et al., 2012	Columbia	To describe the barriers that Colombian health care personnel reported in identifying survivors of DVA and their proposed solutions to improve detection of DVA in the health care setting.	Mixed methods Semi-structured interviews	Naturalistic Inquiry	Obstetrics/gynaecology Internal General Emergency	**27** Doctors Nurses Social workers Psychologists 67% women 33% men	Mean 13 years	74%
Beynon et al., 2012	Canada	To identify barriers and facilitators to asking about DVA among a large, randomly selected sample of nurses and physicians in specified areas of practice where abused women are likely to present.	Mixed methods	Inductive content analysis	Primary Emergency Public health, Obstetrics/gynaecology Maternity Retired	**769** Nurses Doctors 81% women 19% men Age 20–60 years	Not stated	38%
Black et al., 2010	USA	To identify recommended practices of children exposed to domestic violence, as reported by practitioners.	Qualitative individual and group interviews	None stated	Public health Emergency DVA services Mental health	**22** Doctors Nurses Social workers, Managers Academic experts Youth counsellors Advocates 90% women 10% men	Not stated	Not stated
Chang et al., 2009	UK	To explore in more depth the experiences and perspectives of different health professionals regarding what they considered to be necessary to assist them in helping women experiencing DVA.	Qualitative semi-structured interviews and focus groups	Grounded Theory and Triangulation	Obstetrics/gynaecology Midwifery Internal	**24** Doctors Nurses Medical assistants Social workers 88% women 12% men Age 27–58	Not stated	Not stated
Colarossi et al., 2010	USA	To expand current knowledge by comparing licensed family planning service providers (advanced practice clinicians and social workers) and unlicensed ones (health care assistants) who work in a setting guided by an institutional policy and procedure for intimate partner violence screening.	Mixed methods, including focus groups	Grounded Theory	Family planning	**64** Practitioners Social workers	Range 5 - > 10 years	48%
Eddy et al., 2008	USA	To describe one town and gown partnership established to address the health disparities of women experiencing DVA and the children who are exposed to that violence.	Mixed methods Focus groups	Content analysis	Prenatal	**23** Nurses Social workers Lay people	Not stated	100%
Eustace et al, 2016	Australia	To identify midwives’ experiences in relation to screening, ongoing referral and support for women who positively disclose about DVA.	Qualitative semi-structured interviews	Thematic analysis	Midwifery	**21** Midwives 95% women 5% men	Not stated	100% minimal
Evanson, 2006	USA	To describe the unique challenges and opportunities experienced by rural home-visiting PHNs when working with families where DVA was occurring.	Qualitative semi-structured interviews	Descriptive phenomenology	Rural public health	**7** Nurses 100% women	Mean 13 years	100%
Fay-Hillier, 2016	USA	To explore the experiences, views and perceptions of registered nurses working in emergency departments with regard to screening for DVA.	Qualitative semi-structured interviews	Phenomenology Social cognitive theory	Emergency	**21** Nurses 81% women 19% men Age 24–60	Not stated	19% minimal
Goff et al., 2003	USA	To investigate the skills, beliefs, and expectations about screening for domestic abuse among physicians, dentists, and nurse practitioners from a border community in southwest Texas.	Qualitative semi-structured interviews	None stated	Multiple settings	**15** Doctors Dentists Nurses 40% women 60% men	Not stated	Not stated
Goicolea et al., 2015	Spain	To develop a programme theory that seeks to explain how, why and under which circumstances a primary health care team in Spain learned to respond to DVA.	Qualitative semi-structured interviews	Realist evaluation	Primary	**17** Doctors Nurses Paediatricians Midwives Physiotherapists Social workers	Not stated	16% minimal 20% advanced
Haggblom & Moller, 2006	Finland	To explore in depth selected expert nurses’ experiences of the phenomenon of violence against women and the nurses’ roles as health care providers to those women.	Qualitative semi-structured interviews	Constructivist Grounded Theory	Emergency Outpatient Mother-child Mental health	**10** Nurses 100% women	Mean 18 years	Not stated
Henderson, 2001	Canada	To determine how nurses make sense of the interface between themselves, their working environments, and their nursing actions with abused women.	Qualitative focus groups and individual interviews	Social Constructivist	Community health Maternity Emergency Mental health	**49** Nurses 98% women 2% men	Range 6 months-33 years	Not stated
Henriksen et al, 2017	Norway	To gain an in-depth understanding of midwives’ experiences with routine enquiry for intimate partner violence during the antenatal period.	Qualitative Semi-structured interviews	Content analysis	Midwifery	**8** Midwives	Range 3–30 years	40%
Hooker et al., 2015	Australia	To present the findings of a mixed methods process evaluation of the MOVE cluster randomised trial.	Mixed methods Semi-structured interviews	Process evaluation	Community maternal and child health	**23** Nurses Team leaders Nurse mentors	Range 1->20	100%
Hooker et al., 2012	Australia	To explore the breadth of literature on domestic violence screening by nurses in maternal-child practice	Literature review	Scoping review	Community maternal and child health	**17** Papers	n/a	n/a
Husso et al., 2012	Finland	To use frame analysis to explore the ways in which the possibilities for intervening in domestic violence are understood in health care.	Qualitative Focus groups	Frame analysis	Public health	**30** Nurses Doctors Social workers Psychologists 73% women 27% men	Not stated	Not stated
Inoue & Armitage, 2006	Australia	To explore how nurses construct their understanding about domestic violence issues and abused women in relation to their nursing practice.	Qualitative Semi-structured interviews	Grounded Theory	Emergency	**41** Nurses	Not stated	Not stated
Iverson et al., 2013	USA	To assess Veterans Health Administration primary care providers’ perspectives about DVA screening.	Qualitative Semi-structured interviews	Constructivist Grounded Theory	Primary	**12** Doctors Nurses 83% women 17% men	Mean 15 years	8%
Jack et al., 2017	Canada	To develop strategies for the identification and assessment of intimate partner violence in a nurse home visitation program.	Qualitative Focus groups Semi-structured interviews	Content analysis	Community home visiting Mother-child Antenatal	**32** Nurses	Not stated	Not stated
Kirst et al., 2012	Canada	To scope the common elements in the literature about the “critical ingredients” of DVA referral processes in health care settings.	Scoping review	Realist synthesis	Primary Emergency Prenatal	**19** papers	n/a	n/a
LoGiudice, 2015	USA	To glean an understanding of healthcare providers’ experience with prenatal screening for DVA.	Qualitative Meta-synthesis	Meta-ethnography	Women’s health	**8** papers	n/a	n/a
Mauri et al., 2017	Italy	To explore midwives’ knowledge and clinical experience of domestic violence among pregnant women.	Qualitative Semi-structured interviews	Phenomenological-hermeneutical	Midwifery	**15** Midwives	Range 14–35	13%
McCauley et al., 2017	UK	To investigate the knowledge and perceptions of domestic violence among doctors who provide routine antenatal and postnatal care at healthcare facilities in Pakistan.	Qualitative Semi-structured	Thematic framework analysis	Antenatal Mother-child	**31** Doctors Policy-makers 87% women 13% men	Range for doctors 2–10	Not stated
McGarry & Nairn, 2015	UK	To explore the perceptions of emergency nursing staff about the role of a domestic abuse nurse specialist.	Qualitative Semi-structured interviews	Analytic Hierarchy Model	Emergency	**16** Nurses Assistants 94% women 6% men	Range 4 months– 27 years	100%
McGarry, 2016	UK	To explore the experiences of clinical staff in responding to disclosures of domestic violence and to evaluate the role of dedicated nurse specialist.	Qualitative Semi-structured interviews	Analytic Framework	Emergency	**11** Practitioners 100% women	Range 6 months– 30 years	100%
Penti et al, 2017	USA	To explore family medicine physicians’ experiences when interacting with patients whom they know, or suspect, to have perpetrated DVA.	Semi-structured interviews	Grounded Theory	Primary	**15** 33% women 66% men	Up to 10 years	Not stated
Pitter, 2016	Jamaica	To assess midwives’ knowledge and attitudes when encountering gender-based violence in their practice.	Qualitative Participatory action research Focus group	Feminist theory	Midwifery	**6** Midwives Age 28–46	Range 6 months-11 years	0%
Po-Yan Leung et al, 2018	Australia	To explore how doctors perceived the concepts of readiness and preparedness to identify and respond to DVA against female patients.	Qualitative Semi-structured interviews	Thematic analysis	Primary	**19** Doctors 58% women 42% men Age 34–61	Mean 19.5 years	53%
Ritchie et al., 2009	New Zealand	To explore the experiences of emergency nurses one year after launch of routine screening.	Qualitative Single and group interviews	Thematic analysis Triangulation	Emergency	**11** Nurses	Range 1–14 years	100%
Rittmayer & Roux, 1999	USA	To address the methods used by obstetricians/gynaecologists to identify/intervene with patient victims of DVA.	Qualitative Semi-structured interviews	Grounded Theory	Women’s health	**13** Obstetricians/ gynaecologists	Range 20–50	Not stated
Saletti-Cuesta et al., 2018	Argentina	To explore the opinions and experiences of primary care providers regarding DVA.	Qualitative Meta-synthesis	Thematic synthesis	Primary	**46** papers	n/a	n/a
Sormanti & Smith, 2010	USA	To explore barriers to DVA screening and initial steps to address these barriers.	Mixed-methods Focus groups	Thematic analysis	Emergency	**25** Doctors 28% women 72% men	Range 1–3	Not stated
Spangaro et al., 2011	Australia	To understand challenges and enablers of screening and apply this to how health policies become routinised in practice.	Qualitative Focus groups	Normalisation Process Theory Thematic analysis	Antenatal Substance abuse Mental health	**59** Practitioners 90% women 10% men	Not stated	81%
Sprague et al., 2013	Canada	To explore barriers and facilitators to screening for DVA in an orthopaedic fracture clinic.	Qualitative Focus groups Semi-structured interviews	Thematic analysis	Orthopaedics	**22** Surgeons Residents 25% women 75% men	Mean 10 years	Not stated
Sugg, 1992	USA	To explore primary care physicians’ experiences with DVA victims in relation to responding in primary care settings.	Qualitative Semi-structured interviews	Ethnography	Primary	**38** Doctors 37% women 63% men Age 33–58	Mean 15 years	8%
Sunborg et al., 2015	Sweden	To improve the understanding or nurses’ experiences of encountering women exposed to DVA.	Qualitative Semi-structured interviews	Grounded Theory	Primary	**11** Nurses 100% women	Not stated	Not stated
Thi Thanh Nguyen et al., 2014	Vietnam	To explore the underlying beliefs that influence nurses and doctors in screening for victims of DVA.	Qualitative Semi-structured interviews	Planned Behaviour Framework Content analysis	Emergency Outpatient	**19** Doctors Nurses 68% women 32% men Age 18–60	Not stated	Not stated
Varcoe, 1997	Canada	To examine the relationship between the social context of practice and the way in which nurses recognise and respond to women who have been abused.	Qualitative Semi-structured interviews Fieldwork	Critical ethnography	Emergency	**35** Nurses Other practitioners Victim-survivors	Range 4–20	Not stated
Virkki et al., 2015	Finland	To explore how professionals see the possibilities for domestic violence intervention and their role in the process.	Qualitative Focus groups	Frame analysis	Emergency Mental health Maternity Social Work	**30** Nurses Doctors Social workers Psychologists 73% women 27% men	Not stated	Not stated
Watson et al., 2017	UK	To explore primary care psychological therapists’ experiences of working with mid-life and older women presenting with DVA.	Qualitative Semi-structured interviews	Grounded Theory	Primary Mental health	**16** Practitioners 100% women	Range 1–20	0%
Williams et al., 2016	USA	To explore methods by which DVA screening practices are implemented in clinic and emergency settings.	Qualitative Semi-structured interviews	Content analysis	Primary Paediatrics Emergency	**18** Doctors Nurses Managers Medical assistants 72% women 28% men	Not stated	Not stated
Wilson et al., 2016	USA	To explore the experiences of health practitioners who have addressed DVA with migrant and seasonal farm working women.	Qualitative Semi-structured interviews	Phenomenology	Migrant health	**9** Practitioners 100% women Age 29–75	Not stated	Not stated
Zijlstra et al., 2017	Netherlands	To examine factors facilitating and constraining the identification and management of DVA in an emergency department.	Qualitative Semi-structured interviews	Grounded Theory	Emergency	**18** Doctors Nurses Receptionists 56% women 44% men Age 25–60	Mean 5.1	0%
Zink et al., 2004	USA	To examine primary care providers’ awareness about DVA in older women.	Qualitative	None stated	Primary	**44** Doctors Nurses 36% women 64% men	Mean 15.6	Not stated

### Data extraction

Two reviewers (GM and MH) extracted data into a standardised form. Sections included: a description of healthcare-provider-reported facilitators of responding to DVA; a description of healthcare-provider-reported readiness to respond to DVA; author interpretation and direct quotes from participants. The data extraction was discussed by the two reviewers to ensure rigour.

### Thematic synthesis

The data extraction forms were imported into NVivo [18] and thematic synthesis method set out by Thomas and Harden [19] was adopted. The method involves three stages: coding the extracted data using a line-by-line approach; grouping the initial codes into descriptive codes; and generating analytical themes that provide a salient answer to the research question. Unlike the meta-ethnographic approach of Noblit and Hare [20], the thematic synthesis method does not distinguish between first, second and third order constructs. Rather, the method treats the author interpretations and participant quotes as one body of text to be coded using the line-by-line approach. The generation of descriptive codes and broader analytic themes reflects a traditional inductive approach. [21]

One of the lead authors (GM) undertook the line-by-line coding, staying close to the data and preserving the action and language represented in the text. The initial codes were grouped into thirteen descriptive codes. For example, the initial code “finding the nurse specialist role invaluable for both training and support” was grouped with other initial codes about collaborating with other professionals to create a descriptive code “collaborating with specialist professionals.” Using an iterative process through group discussions amongst the authors, the descriptive codes were grouped into analytical themes that provided a narrative to answer the research question. For example, the descriptive code “collaborating with specialist professionals” was combined with the descriptive code “working in a supportive team environment” to create the analytic code “collaborating with a team.” During the descriptive and analytical coding processes, differences in opinion were resolved through discussion. As no date restriction was placed on the date of articles it was important for the authors to think critically about the value of the older papers to the emerging themes. This issue was addressed through a further stage of data analysis whereby the research team, through group meetings, built upon the analytical themes and developed a model of health practitioners’ readiness to address DVA that could be used by contemporary policy-makers and practitioners, but that would also resonate with practitioners trained several decades ago.

### Quality appraisal

Two reviewers (GM and LH) independently appraised each study included in the final sample using a modified version of the *Critical Appraisal Skills Checklist* (CASP) [22] for the singular primary studies and the *Confidence in the Evidence from Reviews of Qualitative research* (CERQual) [23, 24] for the syntheses included in this review. Differences were resolved through discussion or adjudication by a third author (KH).

### Confidence in synthesis findings

Once the analytical themes had been generated, a further stage of quality appraisal was undertaken. This involved applying the CERQual to our own findings. A table was created that set out: the review finding; the studies contributing to the finding; assessment of methodological limitations; assessment of relevance; assessment of coherence; assessment of adequacy; overall CERQual assessment of confidence; and explanation of judgement (Table 2).

**Table 2:** Assessment of confidence in findings.

Theme	Studies contributing	Assessment of methodological limitations	Assessment of relevance	Assessment of coherence	Assessment of adequacy	Overall CERQual assessment of confidence	Explanation of judgement
Having a commitment	20 papers [25–44]	No or very minor methodological limitations (13 studies with no or very minor; four with minor; two with moderate; one systematic review)	No or very minor concerns about relevance (13 papers explored facilitators or readiness; all papers address health practitioners in health settings)	No or very minor concerns about coherence (16 out of 20 papers demonstrate no deviant cases or conflicting data)	Minor concerns about adequacy (20 studies contributed to this theme; 13 offered thick data; seven studies offered thin data)	Moderate confidence	This finding was graded as moderate confidence as it is likely that the finding is a reasonable representation of health practitioners’ readiness to address DVA.
Adopting an advocacy approach	26 papers [27, 28, 30, 32–38, 40–42, 45–57]	No or very minor methodological limitations (17 studies with no or very minor; six with minor; one with moderate; two systematic reviews)	No or very minor concerns about relevance (20 out of 26 papers address facilitators or readiness; all studies address health practitioners in health settings)	No or very minor concerns about coherence (20 out of 26 studies demonstrate no deviant cases or conflicting data)	No or very minor concerns about adequacy (26 papers contributed to this theme; 20 offered thick data; six offered thin data)	High confidence	This finding was graded as high confidence as it is highly likely that the finding is a reasonable representation of health practitioners’ readiness to address DVA.
Trusting the relationship	30 papers [26, 29, 30, 32–36, 38, 39, 41–43, 45–47, 49, 51–63]	No or very minor methodological limitations (18 studies with no or very minor; seven with minor; two with moderate; three systematic reviews)	No or very minor concerns about relevance (29 out of 30 papers explored facilitators or readiness and all papers addressed health practitioners in health settings)	No or very minor concerns about coherence (26 out of 30 papers demonstrate no deviant cases or conflicting data)	No or very minor concerns about adequacy (30 papers contributed to this theme; 27 offered thick data; three studies offered thin data)	High confidence	This finding was graded as high confidence as it is highly likely that the finding is a reasonable representation of health practitioners’ readiness to address DVA.
Collaborating with a team	27 papers [26, 28, 29, 31, 33–36, 38, 39, 42, 45–50, 56–58, 60, 63–68]	No or very minor methodological limitations (18 studies with no or very minor; seven with minor; two systematic reviews)	No or very minor concerns about relevance (20 out of 26 papers addressed facilitators or readiness; all papers addressed health practitioners in health settings.	No or very minor concerns about coherence (26 out of 27 papers demonstrate no deviant cases or conflicting data)	No or very minor concerns about adequacy (27 papers contributed to this theme; 23 offered thick data; four offered thin data)	High confidence	This finding was graded as high confidence as it is highly likely that the finding is a reasonable representation of health practitioners’ readiness to address DVA.
Being supported by the health system	35 papers [25–31, 33–36, 38–43, 45–47, 49–53, 56, 57, 59, 64–70]	No or very minor methodological limitations (20 studies with no or very minor; eight with minor; four with moderate; three systematic reviews)	No or very minor concerns about relevance (23 out of 30 papers addressed facilitators or readiness; all papers addressed health practitioners in health settings)	No or very minor concerns about coherence (29 out of 35 papers demonstrate no deviant cases or conflicting data)	No or very minor concerns about adequacy (35 out of 46 papers contributed to this theme; 27 offered thick data; eight offered thin data)	High confidence	This finding was graded as high confidence as it is highly likely that the finding is a reasonable representation of health practitioners’ readiness to address DVA.

Assessment of **methodological limitations** involved assigning a limitation rating to each study as in the CASP appraisal, including: no or very minor concern; minor concern; moderate concern; and serious concern. To ascertain a measure of methodological confidence in each synthesis theme, the papers contributing to the theme were rated and the percentage of papers that had no, or very minor methodological limitations was calculated. It was decided that if 50% of papers contributing to the theme were rated as having no or very minor methodological limitations, then the overall assessment of methodological limitation related to the theme would be rated as no to very minor.

Assessment of the **relevance** of synthesis findings involved exploring two “measures” of relevance, including: whether a paper addressed readiness or facilitators overtly; and whether the majority of participants in a study were health practitioners in health settings. Synthesis findings were assessed as having no or very minor concerns if most papers contributing to the theme addressed readiness or facilitators overtly and the majority of participants and settings in the papers were health practitioners in health settings.

Assessment of **coherence** of review findings involved examining the fit or deviance between a review finding (theme) and the data contributing to the finding. Synthesis themes were assessed as having no or very minor concerns about coherence if the majority of the papers contributing to the theme had no cases of data that deviated from that which supported the theme. Assessment of **adequacy** of review findings involved two considerations: how many papers out of the whole sample contributed to the theme; and the thickness or ‘richness’ of the data that supported the theme. ‘Rich’ data provides enough detail to understand meaning and context. Synthesis themes were assessed as having no or very minor concerns if the papers contributing to the theme constituted more than 50% of the overall sample of papers and if over 50% of that contributing data was sufficiently thick.

A level of confidence was assigned to each of the findings, ranging from very low confidence whereby it is not clear if the finding is a reasonable representation of the phenomenon of interest, to high confidence whereby it is highly likely the finding is a reasonable representation of the phenomenon of interest. That is, no or very minor concerns meant high confidence. An overall assessment of confidence in the synthesis findings was generated by weighing up the methodological limitations, relevance, coherence and adequacy of the papers contributing to each theme. An assessment of moderate confidence was given to themes that had been assessed as having minor concerns on any of the CERQual elements. If synthesis findings had been assessed as having no or very minor concerns on any of the CERQual components, then the theme was assessed as high confidence.

## Results

Forty-seven papers were included in the review. These included forty-one primary empirical studies published in 35 journals, four systematic literature reviews [31, 55, 57, 59] and two doctoral theses (Table 2). [25–28, 30–36, 38–62, 64–72] The primary empirical studies included data from 1,744 practitioners about their perceptions of what enhances readiness to address DVA. The health practitioners in the primary studies had between four months and 50 years of professional experience across specialisations including: emergency medicine; primary care; intensive care; obstetrics/gynaecology; maternal and child health; family planning; prenatal and antenatal medicine; mental health; orthopaedics; paediatrics; dentistry; and allied health. Of the studies, 16 originated in the United States, six from Australia, five each from the UK and Canada, three from Finland, two from Columbia and one each from Jordan, Spain, Norway, Italy, New Zealand, Argentina, Vietnam, the Netherlands, Jamaica and Sweden. The four reviews all had different objectives to the aim of our review but had some findings relevant to readiness. Hooker et al explored the breadth of literature about DVA screening by maternal and child health nurses, and Kirst et al reviewed the “critical ingredients” of DVA referral processes in health care settings. Further, LoGiudice et al aimed to understand the experience of health care providers in prenatal screening for DVA, and Saletti-Cuesta explored the opinions and experiences of primary care practitioners in relation to DVA. The systematic literature reviews were included because there was very little overlap between the primary studies in our sample and those in the reviews with only one study overlapping. [45]

Our qualitative meta-synthesis resulted in the development of five themes representing health practitioners’ perception of readiness to address intimate partner violence. The five themes are: *Having a commitment; Adopting an advocacy approach; Trusting the relationship; Collaborating with a team; and Being supported by the health system*. These themes reflect for the most part how health practitioners feel ready to address DVA for survivors, as there was only one study discussing responding to men who use violence as patients, none on male victims [45] and one that concentrated on children’s experience of DVA. [50]

### Having a commitment

Health practitioners highlighted that readiness to address DVA is influenced by having a personal commitment to the issue (across 20 papers). This commitment arises through having a personal experience of DVA in their home life or family or through adopting a feminist-like or human-rights-informed ideological conceptualisation of DVA. Further, a commitment can arise through possessing a strong belief that the best interests of children must be held as paramount.

Doctors, nurses and midwives across emergency medicine and primary care expressed the view that personal experience informed their commitment to addressing DVA (see Table 1). In a Canadian mixed-methods study [26] involving 769 doctors and nurses, the authors found that a personal experience of DVA facilitated healthcare providers’ readiness to address the problem. One nurse commented: “My personal experience with abuse provides me with a comfort level, knowledge of the system and a desire to support and empower women.” [26] p8 Another nurse said: “The fact that I have been a victim of domestic violence and abuse makes it easier for me to identify women who are experiencing a similar situation.” [26] p8

A wide range of practitioners, including general practitioners, midwives, obstetricians/ gynaecologists and surgeons talked about the influence of a feminist-informed ideological commitment to addressing DVA in their practice. In a Spanish study [28] involving 17 primary care health practitioners, the authors found that there was a group of professionals who held feminist-like views about empowering women. These professionals undertook a process of continuous self-learning about DVA and inspired others to do the same. Leung et al. [32] p520, in their study of 19 primary care doctors, found that the majority of participants were motivated by an understanding that DVA is a violation of human rights: “The majority of participants emphasized that they would be there for patients experiencing DVA and point out that DVA is a violation of human rights.”

Providers also talked about how adopting a best-interest-of-the-child lens enhanced their readiness to address DVA. A study undertaken in Finland. [41] p15 indicated that practitioners felt adopting a children’s lens enabled them to address instances of DVA, even when there were barriers like the presence of the perpetrator. One practitioner stated:

In these situations, the [potentially abusive] husband is also present, so the question is, in what situation can it (suspicion of violence) be brought up, and how. In my opinion, the baby and the children provide a way.

Overall, this synthesis finding indicates that practitioners’ readiness to address DVA is influenced by their personal belief systems. These systems can be shaped by their personal experience or by feminist, human rights or best-interests-of-the-child ideological frameworks. A personal commitment informed by the belief that DVA is unacceptable sets the intention of health practitioners to intervene when they encounter DVA in their practice. The findings in this theme may be particularly relevant for nurses working in emergency settings as many studies were done on this population. The authors had moderate confidence in this finding, according to the CERQual method, which means that it is likely that the finding is a reasonable representation of health practitioners’ readiness to address DVA.

### Adopting an advocacy approach

The personal commitment to DVA issues might set the pathway for health practitioners to take action by adopting an advocacy approach to addressing DVA, involving helping survivors on a pathway to safety and wellbeing. Twenty-six papers contained data that contributed to this theme (See Table 1) from a wide range of health professionals (including primary care doctors and nurses, maternal and child health nurses, mental health workers and obstetrician/ gynaecologists). Health practitioners historically felt they needed to fix the problem but express the understanding of the need for an advocacy approach to DVA, working as an ally with patients.

More than 30 years ago several studies addressed this issue. A US study [46] explored the way 13 obstetrician/gynaecologists addressed DVA and found that in order for health practitioners to relinquish the need to fix the problem of DVA, they had to abandon the traditional model that had underpinned their medical degrees. Instead of viewing success as the woman leaving the relationship, they began to see having contact with a woman as success in itself. The authors indicated that the obstetrician/gynaecologists who gave up the need to directly change the circumstances of victims and were able to acknowledge the limitations of their role, assisted their readiness to do the work. Relinquishing the traditional role of “fixer” is echoed in another US study [37] p3160 of 38 general practitioners. An ethnographic approach was employed in this study to explore primary care doctors’ experiences identifying and responding to DVA. The author found:

[General practitioners] perceived their role as validating a patient's feelings, discussing safety issues, and referring patients to appropriate resources. They also saw the time frame for change as a prolonged course and were not concerned with the idea of a quick fix.

Health practitioners talked about adopting an approach to practice that focuses on engaging women in the journey to safety. [41] This practice involves assuming a non-judgemental temperament and using active listening skills to engage with women. In a US study [54] p2221 involving 32 nurses in a home visitation program, the authors found that nurses use open-ended questions and validation of women’s experiences to engage them in conversations about DVA. The authors state:

The nurses emphasised the importance of conversing with clients rather than a traditional approach of ‘telling’ or ‘educating’ clients about what to do.

The approach embraced by health practitioners emphasises that women are the experts in their own lives. An Australian study [34] p520 of 19 general practitioners exploring about their perceptions of readiness to respond to DVA indicated that health practitioners need to be guided by women’s readiness to address their situations. In that study, one participant said:

Is she really not ready to even acknowledge [the DVA] or does she acknowledge it but she doesn’t want to do anything or is she getting ready to do something, and you give her different support and different help according to where she is along that road.

Overall, health practitioners begin to act on their intention to address DVA through relinquishing the traditional approach to management and adopting women-centred practice that avoids victim-blaming and supports the act of listening to women. This finding may be most relevant for a variety of health practitioners working in primary care settings. The authors had high confidence in this finding according to the CERQual method which means that it is highly likely that the finding is a reasonable representation of health practitioners’ readiness to address DVA.

### Trusting the relationship

Clinicians experience of the professional relationship with their patients underpins health practitioners’ readiness to address DVA. Clinicians saw their clinical role as ideal for responding to survivors as they often are in a position of trust, even when they see the patient for the first time or particularly in models of care that allow clinicians to talk to patients over time. Thirty papers contributed data about this theme (See Table 1). Practitioners talked about how members of the public have an intrinsic trust in health professionals and that they received positive feedback from women when they broach the topic of DVA. In addition, they also discussed how their clinical role enables them to form clinical relationships with victims, building trust over time.

A UK study involving 16 health practitioners in an emergency department setting [60] found that practitioners perceived their role as placing them in a position of trust that invites people to confide in them. Further, a Canadian study [36] p7 involving focus groups with 20 surgeons indicated that an inherent public trust in the medical profession facilitates practitioners’ readiness to address DVA. One participant said:

I’m sometimes surprised at how open and forthcoming patients are in the short time you get to know them the things that they’ll tell you… there is a sort of inherent trust in the medical profession.

This sense that the clinical role is ideal for intervening in DVA was reinforced by the positive response that health practitioners received from women. Practitioners in an Australian study [47] p136 described how women are positive and grateful when asked about DVA and that this acts as an enabler for the practitioners. One participant observed that when addressing the issue of DVA with women that: “It’s almost like a flood gate has opened, that “[health practitioner has] now given [victim] the opportunity.” Another participant reflected about asking women about DVA: “How many other times in their life have they had the question asked? And they’re like, ‘I’ve been waiting for someone to ask me and no one asks me.’”

A New Zealand study [35] p18 involving single and group interviews with 11 emergency department nurses indicated that nurses were encouraged by receiving positive feedback from women. One participant stated: “I think the feedback you get too, because the number of times I’ve routinely questioned and more often than not the woman’s said, I think that’s really good what you’re doing.” A clinician in a study by Henriksen et al., [30] p5 echoed the sentiment that positive feedback is an enabler for addressing DVA: “And that was what I discovered, when we dare to ask, when we dare to open up and perhaps demonstrate that we can handle this, the answers, then they say yes. Much more often than what I would have thought.”

Health practitioners’ experience, that the clinical role is ideal for responding to DVA- is further strengthened by the ongoing relationships they can build with victims and their families. Midwives in an Australian study. [52] p507 talked about how building a relationship with women over time acts as an enabler for addressing DVA. One midwife said: “I’ve now got the advantage of time and the advantage of continuity of care… Asking those questions can be done in a much more collegial way.” Another stated: “Because you can get to know them and can really champion their cause…it’s such a difficult topic to broach when you first meet someone.” The importance of the relationship held true for addressing women victims as well as male perpetrators. In a US study [63] p243 general practitioners reported that a strong relationship with male patients made it easier to raise the issue of DVA perpetration:

I think the conversation [about perpetration] went smoothly because I had been there for him in tough times.

Rural nurses in a US study [58] p11 identified an advantage that they have over non-rural nurses. They note that living in a rural community enabled them to form and maintain strong relationships with women because they have more opportunities to have contact with women in both clinical and non-work settings. One nurse said:

The women who I visit in the home, I will see them in a different setting as well… I see them in the grocery store. I see them out in the parking lot with their boyfriend and their kids.

Overall, health practitioners perceive their clinical role as ideal for addressing DVA amongst their patients. They understand that the public has great trust in the health profession and they are buoyed by women’s positive reactions when asked about DVA. They also recognise the importance of continuity of care through forming strong relationships in their ability to effectively respond to DVA. The authors had moderate confidence in this finding according to the CERQual method which means that it is likely that the finding is a reasonable representation of health practitioners’ readiness to address DVA. The findings in this theme may be relevant for a wide range of health practitioners working across community-based and hospital-based settings.

### Collaborating with a team

A further action undertaken by health practitioners to enhance their readiness to address DVA involves their collaboration with their team members internal to their organisations and with specialist professionals outside their team. Practitioners spoke broadly about the comfort and support that these collaborations provide. Twenty-seven studies included material that gave rise to this theme (See Table 1).

Doctors and nurses in primary care identified the importance of having a team behind them when addressing DVA. In a study of 12 primary care providers treating female veterans in the United States [45] p827, the authors found that the team-based approach facilitated a response to DVA. A participant in the study said:

I think the team can facilitate [DVA screening] because if you have a patient you’re concerned about…I think having a team that is on board with you in that feeling it’s important, you have people to go to and ask about resources.

In a qualitative meta-synthesis [57] p414, the authors indicated the importance of interdisciplinary teams that provide emotional support and collective care strategies in the primary care setting. One health practitioner said: “It’s only through sharing the experience and talking about it and getting the support of your colleagues, then it eases the burden to deal with it.”

Other health practitioners talked about how team members were not only important for emotional support but for inspiring others to address DVA. Goicolea et al. [28] found that health practitioners achieved a sense of self-confidence and self-efficacy through their daily engagement with a small group of professionals who were highly committed to addressing DVA. The practitioners also described a monthly group meeting in which they could debrief about their experiences of addressing DVA in the workplace, including discussion of their feelings about dealing with DVA and any trauma that emerged from working with DVA.

Readiness to address DVA was enhanced not only through having a supportive team environment, but through collaborating with specialist professionals. This was particularly true for clinicians in organisations where they had access to a specialist DVA nurse. In a UK study [66] involving 11 clinical staff in a National Health Service setting, the author found the training and support provided to the clinicians by the DVA nurse specialist had been invaluable.

Health practitioners also talked about their strong reliance on other specialist DVA professionals in their clinical settings. The health practitioners in Spangaro, Poulos and Zwi’s [47] p136 study talked about the utility of having a social worker on call who could assist with consultations involving DVA and help alleviate clinicians’ sense of needing to fix the problem. One participant said:

I was able to ring the social worker after the woman accepted, and she dropped everything and came immediately. That made me straight away feel, “Oh, it’s okay”. All I had to do was ask and respond in a really supportive way.

Similarly, in a UK study. [64] p198 of 24 health practitioners in various settings, the authors found that obstetrician/gynaecologist participants perceived the value of collaborating with social workers:

I talked to the social worker and we got hooked up with a number to call and [my patient] did counselling on the phone because she was homebound.… So, it really helped her decide what she wanted to do and take the steps to do it in a safe way.

Overall, health practitioners’ readiness to respond to DVA is supported through having a strong team approach to addressing DVA, including collaborating with professionals who have specialist knowledge about abuse and social sector services. The authors had high confidence in this finding according to the CERQual method which means that it is highly likely that the finding is a reasonable representation of health practitioners’ readiness to address DVA. The finding may be most relevant for nurses in emergency and primary care settings.

### Being supported by the health system

Readiness to address DVA is fully realised when provider intention and actions are supported by a strong health system equipped to manage DVA. This was the largest theme of the five, with 35 papers contributing data (See Table 1). Health practitioners talked about needing the health system to support them through: upskilling in how to address DVA; making asking about DVA routine; allowing time to do the sensitive work with patients; and creating an authorising organisational environment. Reflective practice and monitoring with feedback so that health practitioners can see what they are doing and improve was also suggested by some practitioners.

Many healthcare practitioners talked about the importance of being trained in how to identify and respond to DVA in their clinical setting. For example, in a Columbian study [49] p257 involving 27 healthcare providers from different specialisations, the authors found that the majority of participants in their sample wanted more training about DVA. This finding that training is essential for enhancing practitioner readiness to address DVA is supported by Saletti-Cuesta, Aizenberg and Ricci-Cabello’s [57] systematic review of 46 qualitative studies. The review indicated that training and continuing education were important facilitators for addressing DVA. Further, an Italian study [56] p501 of 15 midwives suggested that continuing education is essential for improving readiness to address DVA. One midwife stated:

It would be useful, now that I’ve graduated, to participate in courses about this, to improve my knowledge and skills in detecting and dealing with domestic violence.

Health practitioners also spoke about needing resources to assist their response to DVA amongst their patients. In a US study [51] p240 involving 64 family planning health providers, the authors found that participants wanted referral materials like discreet cards and brochures. In the same study, participants wanted practice guidelines setting out how to respond during a consultation. One participant said: “I need some helpful scripts or specific sentences to say to patients, because I don’t know what to say when they tell me they are being abused.” Likewise, dentists, doctors and nurses who participated in Goff et al.’s [53] study in the US indicated that they needed guidance in how to broach the subject of DVA with patients and that this should be part of their DVA training. Further, the primary health care providers in a US [45] study called for clinical tools and resources to assist them with addressing DVA.

As well as needing to be upskilled to identify and respond to DVA, health practitioners also spoke about the need for broaching the subject of DVA with women to become part of routine practice. This does not necessarily mean that health practitioners called for the formal screening of all women in their clinical settings but rather that asking about DVA should be part of a normal assessment process. Participants in a Colombian study [49] talked about the need to ask about DVA as part of the general shift to inquiring about mental health to complete comprehensive patient histories. General practitioners in Sugg’s [37] p3160 US study noted the value of the way that some of their colleagues dealt with DVA. The author said:

There were two physicians who stood out from the rest because of their level of comfort in dealing with domestic violence… They had a comfortable, neutral, business-as-usual approach to asking questions about violence.

This everyday approach to asking about DVA is echoed in an Australian study [47] p135 in which health practitioners identified screening questions as enablers of addressing DVA. One participant said: “So to be really simple about it, you get the folder, you turn the first page, you ask the questions. It’s part of the intake process.”

The process of enquiry carried out in a routine way could be enabled through a supportive organisational environment. In a US study. [25] p60 involving 19 practitioners from a range of specialities, the authors found that providers who routinely asked about DVA worked in organisations in which managers support and encourage the practice. One such practitioner said:

I feel our department is very supportive of that. Our management, directors…[The screening question] is part of the section that is referred to as the essential elements.

Addressing DVA through inquiry could also be enhanced through another organisation factor: clear protocols and policies regarding abuse issues. In the paper by Inoue and Armitage [33] p318-319 involving 41 emergency nurses in Australia and Japan, the authors stated:

When [policies and procedures] were put in place nurses were clear as to what was expected of them and what services were available to them when they encountered women who had been abused.

This sentiment was echoed by Goicolea et al.: [28] p8

The policies did play a role in providing legitimacy for their work and were considered a strong sign of recognition (mechanisms of legitimisation and recognition).

The use of organisational-level policies and procedures could, in turn, be supported by an authorising legal or societal environment. Health practitioners noted that public health campaigns involving the media could help increase community awareness of DVA. Further, doctors and nurses in Beynon et al.’s study suggested that media-based campaigns could help to normalise routine inquiry about DVA so that women do not feel alarmed when asked. This sentiment was also expressed by dentists, doctors and nurses in Goff et al.’s [53] p144 study. The authors stated:

The idea that there should be an increase in general awareness of abuse, including addressing the problem more universally or routinely in a clinical setting, was also a common theme expressed.

Overall, this thematic category captures health practitioners’ perception that DVA training and a strongly supportive health system is essential to their readiness to respond to DVA. Organisational and societal support was required to upskill health practitioners and to enable routine inquiry about DVA as part of the standard assessment process. Further, policies and procedures were needed to anchor this approach to DVA in everyday practice. This finding may be most relevant for a variety of health practitioners in emergency and primary care settings. Addressing DVA in health settings could be further enhanced through a legitimising social environment more broadly. The authors had moderate confidence in this finding according to the CERQual method which means that it is likely that the finding is a reasonable representation of health practitioners’ readiness to address DVA.

**Fig 2:**
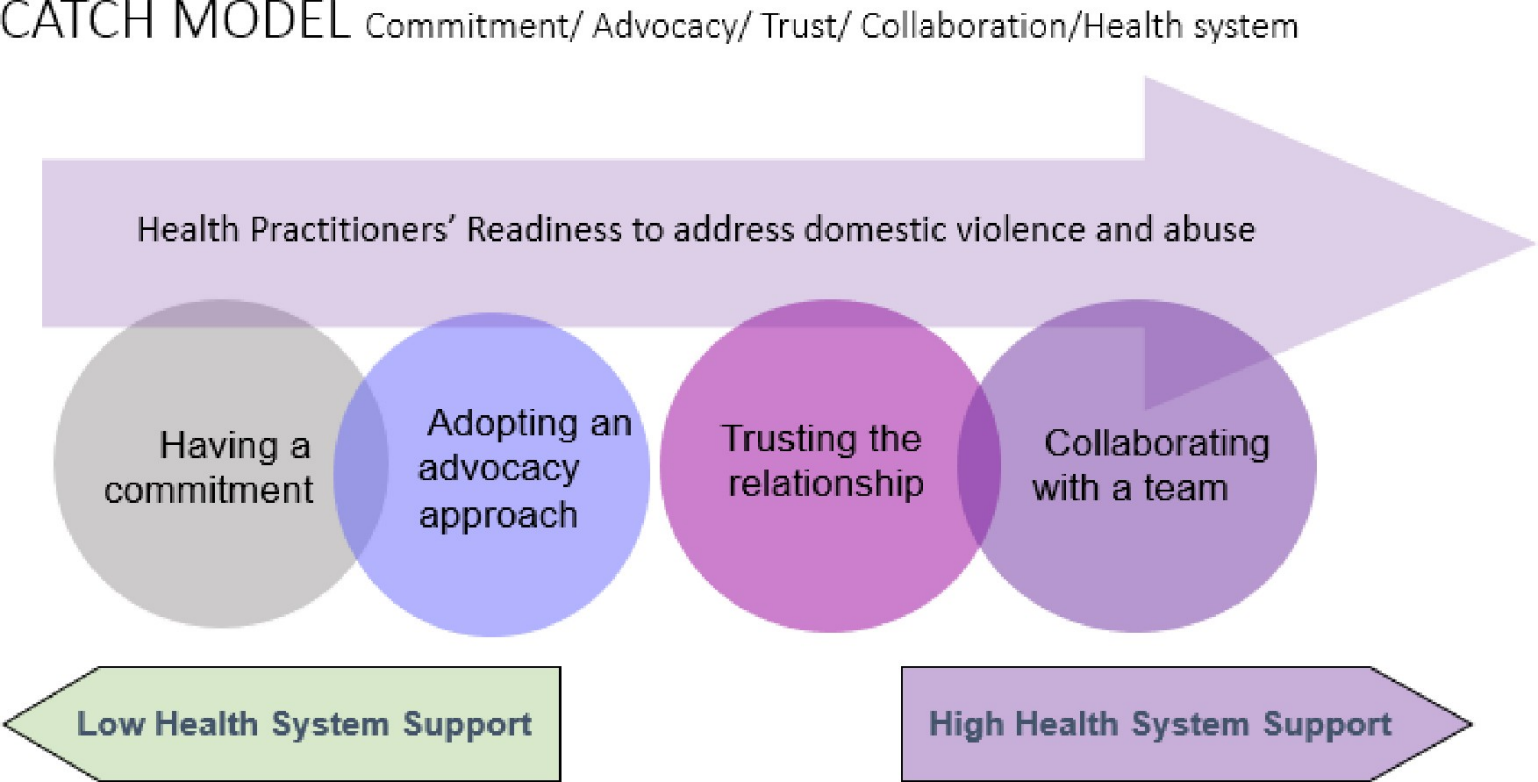
Health practitioner’s readiness model: The CATCH model.

In summary, we have synthesised these findings into a model, which we have called the CATCH Model -Commitment, Advocacy, Trust, Collaboration, Health system support (Fig 2).

## Discussion

This systematic qualitative meta-synthesis found five emerging themes relating to health practitioners’ readiness to address intimate partner violence: *Having a commitment; Adopting an advocacy approach; Trusting the relationship; Collaborating with a team; and Being supported by the health system*. There has been less attention paid to internal belief and value systems of practitioners that might motivate them to undertake the complex work of identifying and responding to DVA. *Having a commitment* through personal experience is demonstrated in an Australian study where survivor staff were shown to ‘go the extra mile’ by attending training and providing clinical practice of a high standard. [73] Motivation through a rights-based belief system [34] is also an untapped area to assist practitioners to become ready to do this work. This area could be explored more as many survivors including Indigenous Peoples and other socially disadvantaged ethnic minorities are further entrapped by health inequities of poverty, racism, colonisation and discrimination because of sexuality, gender and disability. [74, 75] *Adopting an advocacy approach* with patients has been called for by survivors, practitioner and organisations for over a decade. [6, 76] Practitioners need to be able to ‘let go’ of the control of the consultation if they are going to be able to address DVA and focus on listening actively to the survivor. [3] Papers that discussed the need to relinquish the traditional medical model of fixing a patient’s problem were more dated than papers contributing to the other themes. The authors acknowledge that this theme reflects training and attitudes from previous decades but that health practitioners who trained during that time may not have been exposed to current patient-centred care practice.

Our findings around *Trusting the relationship* whereby the clinical setting is seen by health practitioners as an ideal place for this work is supported by the World Health Organisation guidelines. [3] Clinical experience of actually engaging in the work over time has been shown to enable readiness to address family violence. [34] Further, hearing about positive outcomes for patients can help reinforce to clinicians that they are in the best place to deal with DVA. [47] We know from evidence to change health practitioner behaviour in other areas that these feedback loops and reflexive monitoring are a key way to support improvements in practice. [77, 78] However, it must be acknowledged that the health care system may not be a place of trust from some patients viewpoints, particularly marginalised populations. [74, 75]

Health practitioners cannot do this alone though. *Collaborating with a team* for support and for more specialised advice is a basic tenet of practice in any area. [77] In another study, a key part of how work gets done in sexual violence and mental health services was the need for relationship building within and across teams. [79] In this case study, staff connection within teams, within the hospital and with external services through opportunities to talk together developing a shared understanding of their roles and integrated coordinate care enhanced how patient care was delivered.

Finally, transforming our health systems is evident in the theme: *Being supported by the health system*. [80] Integrated coordinated care for intimate partner violence requires support through leadership, policies, protocols, champions, infrastructure, environments, data systems for feedback and a supportive culture. This is the first step needed in any process of reform, so that we are not setting up practitioners to fail. [80] Often programs provide DVA training without having a systems approach that acknowledges the varying levels of readiness of practitioners and teams to undertake this challenging work.

The CATCH Model -Commitment, Advocacy, Trust, Collaboration, Health system support (Fig 2) from our findings is the first model of readiness of health practitioners published in the literature. We have applied this model to different Stages of Change or readiness to undertake the work (Table 3). There has been work on organisational readiness checklists by World Health Organisation, [80] but we could not find a conceptual model of readiness for health professionals to address DVA.

**Table 3:** Readiness to address domestic violence and abuse (DVA) and tailored responses to different stages of change.

Stage of Change	Response by health practitioner
**Pre-contemplative** Does not think that addressing DVA is their role	**Encourage commitment to the issue.** Suggest possibility of a connection between patient’s health issues and DVA and that the health setting is placed well and equipped to address this complex issue.
**Contemplation** Has identified a problem or need to address DVA but remains unsure about whether they are able to undertake the work	**Assess needs of practitioner to provide an advocacy approach with patients.** Point out that the workplace is available to support them on journey to addressing domestic abuse through training and resources available e.g. support for survivor staff is in place.
**Preparation/decision** Catalyst for change has arisen (saw a particular patient, attended training, heard a story about personal experience in friends or family)	**Explore issues of clinical experience and level of trust in relationship with patients to undertake this work.** Respect decision about what they want to do (asking patients routinely in antenatal care, wearing a lanyard, attending training, documenting better in files, speaking out about DVA to staff as survivor).
**Action** Plan devised in the previous stage is put into action	**Ensure collaboration with a team both internally and externally is strong.** Offer support to carry out plan and ensure workplace support is in place e.g. policies, procedures, posters, tools
**Maintenance** Commitment to above actions firm	**High system support with feedback loops from patients are strong** Celebrate whatever they have managed to do and support their actions.
**Returning** May feel very frustrated and unable to address DVA as they would like. Reasons include life stressful, no access to resources, system not supportive.	**Engage in advocacy for system support.** Need to keep engaged even if they are unable to address DVA in their workplace. Reassure that this pattern is common and may need to wait until there are higher system supports in place.

### Strengths and limitation

The strengths of this qualitative meta-synthesis are that, to our knowledge, this is the first systematic review of qualitative evidence about the readiness of health practitioners to identify and respond to DVA, including to women, children and men. It brings together a vast literature about the factors that facilitate health practitioner readiness to respond to women but is limited in response to men or children. The synthesis uses a rigorous qualitative systematic review methodology, including the screening of all papers by at least two researchers. Further, strengths include the application of the CERQual tool to the findings of the review, providing an overall indication of confidence in each theme. Limitations include that many papers addressed facilitators to the work rather than the larger concept of readiness including self-efficacy, emotions, motivations and attitudes directly. That is, the research involved asking health practitioners about what facilitates their ability to address DVA rather than about the concept of what would enable their readiness to do the work. However, it was decided that papers that addressed facilitators would be held as equivalent to papers that addressed readiness. Also, it is true to say that different themes were supported more or less by different sets of health practitioners, making it difficult to declare that the model of readiness developed through the findings pertains to all health practitioners in the same way. Another limitation was that most of the studies were carried out in high-income, developed countries. This means that the finding may be less applicable to lower-income countries with less well-developed health systems. However, overall the quality of the papers was good and the model developed, in our analysis, contributes significantly to the evidence about health practitioner readiness to identify and respond to DVA.

### Implications and conclusion

What does a ‘*ready*’ health practitioner look like? They are motivated to make a difference, they know how to do an approach based on advocacy, they feel they are likely to succeed as the health setting is a good place to identify and respond to patients, they have received encouraging feedback, they work with others and they are strongly supported with ongoing DVA training, clinical protocols, tools and leadership in the health system. The CATCH Model (Fig 2) and the Stages of Change model (Table 3) may be helpful for trainers to inform educational programs about the best responses to levels of readiness to undertake this work. It will also assist managers and program leads on DVA to understand strengths and resistance in the workforce. We suggest that a shift in the focus of health practitioner training to address the ‘readiness’ factors identified in this review rather than just on ‘barriers and facilitators’ as has been done previously as this may increase practitioner confidence and capability to do the work. Further research is needed about applying the CATCH model in programs to see if it assists transformation of clinician’s readiness to address DVA.

## Supporting information

S1 Table Search terms used in Ovid. (DOCX)
