# Supplementary material for: Health practitioners' readiness to address domestic violence and abuse: A qualitative meta-synthesis
Source: PLoS One. 2020 Jun 16;15(6):e0234067. doi: 10.1371/journal.pone.0234067 (PMC7297351; doi:10.1371/journal.pone.0234067)
Supplement: S1 Table — (DOCX) [file pone.0234067.s001.docx]

**SI Table. Search terms used in Ovid**

| Search terms |
| --- |
| Battered women/ or domestic violence/ or spouse abuse/ or  marital rape/ or (abuse$ adj3 wom#n).tw or (abuse$ adj3 spous$).tw or (abuse$ adj3 partner$).tw or (abuse$ adj3 (wife or wives)).tw or (batter$ adj3 (wife or wives)).tw or (batter$ adj3 wom#n).tw or domestic violence.tw or family violence.tw or dating violence.tw or (partner$ adj3 violen$).tw or (spous$ adj3 violen$).tw or (gender adj3 violen$).tw or Reproductive coercion.tw |
| exp Qualitative Research/ |
| Phenomenology.mp |
| exp Hermeneutics/ |
| Constructivism.mp |
| interview/ |
| social sciences/ or theoretical orientation/ |
| questioning/ or information seeking/ or interviewing/ |
| Observation/Mt |
| grounded theory/ |
| program evaluation/ |
| Verbal Communication.mp |
| exp personal narratives/ |
| discourse analysis or content analysis.mp |
| sociocultural factors.mp |
| exp Health Attitudes/ or Attitudes/ or exp Client Attitudes/ or exp Consumer Attitudes/ or exp Female Attitudes/ |
| Community Attitudes.mp |
| cultural sensitivity.mp |
| (qualitative or ethno$ or emic or etic or phenomenolog$ or hermeneutic$ or heidegger$ or husserl$ or colaizzi$ or giorgi$ or glaseror strauss or van kaam$ or van manen or constant compar$).ti,ab |
| (focus group$ or grounded theory or narrative analys$ or lived experience$ or life experience$ or theoretical sampl$ or purposive sampl$ or ricoeur or spiegelberg$ or merleau or metasynthes$ or meta-synthes$ or metasummar$ or meta-summar$ or metastud$ or meta-stud$ or maximum variation or snowball).ti,ab |
| ((thematic adj3 analy$) or (content analy$ or field notes or fieldnotes or field record$ or field stud$) or (participan$ adj3 observ$) or (nonparticipan$ adj3 observ$) or (non-participan$ adj3 observ$)).ti,ab |
| (semi-structured or semistructured or structured categor$ or unstructured categor$ or action research or (audiorecord$ or taperecord$ or videorecord$ or videotap$) or ((audio or tape or video$) adj5 record$) or interview* or quasi-experiment* or (case adj stud*)).ti,ab |
| (collaborat* or consultat* or experience or involve* or narrative* or opinion* or participat* or partner* or perspective* or story or stories or view* or voice*).ti,ab |
| self report/ |
| Yarn* |
| Doctor* or nurse* or midwi* or dentist* or psychologist* or (health$ adj3 provider).tw or (healthcare$ adj3 provider).tw or (healthcare$ adj3 worker).tw or (health$ adj3 personnel).tw or (health$ adj3 worker) or (allied adj3 health) |
| exp health personnel/ |
